# A new approach to estimation of the number of central synapse(s) included in the H-reflex

**DOI:** 10.1186/1471-2377-5-13

**Published:** 2005-07-12

**Authors:** Mohammadreza Alavian Ghavanini, Alireza Ashraf, Shahram Sadeghi, Mohammadreza Emad

**Affiliations:** 1 Department of physical medicine and rehabilitation, Shiraz medical school, Zand avenue, Shiraz, Iran; 2 Pain research group, Academic centre for education, culture and research, Iran medical science branch. No 31, Karimkhan Zand avenue, Shahid Hosseini alley, Multidiscipnilary pain clinic. Tehran, Iran

## Abstract

**Background:**

Among the main clinical applications of the H-reflex are the evaluation of the S1 nerve root conductivity such as radiculopathy and measurement of the excitability of the spinal motoneurons in neurological conditions. An attempt has been made to reduce the pathway over which H-reflex can be obtained in a hope to localize a lesion to the S1 nerve root, so the S1 central loop has been suggested. The main goal of this study is the estimation of the H-reflex number of synapse(s) for better understanding of the physiology of this practical reflex.

**Methods:**

Forty healthy adult volunteers (22 males, 18 females) with the mean age of (37.7 ± 10.2) years participated in this study. They were positioned comfortably in the prone position, with their feet off the edge of the plinth. Recording electrodes were positioned at the mid point of a line connecting the mid popliteal crease to the proximal flare of the medial malleolus. Stimulation was applied at the tibial nerve in the popliteal fossa and H, F and M waves were recorded. Without any change in the location of the recording electrodes, a monopolar needle was inserted as cathode at a point 1 cm medial to the posterior superior iliac spine, perpendicular to the frontal plane. The anode electrode was placed over the anterior superior iliac spine, and then M and H waves of the central loop were recorded. After processing the data, sacral cord conduction delay was determined by this formula:

* Sacral cord conduction delay = central loop of H-reflex – (delays of the proximal motor and sensory fibers in the central loop).

**Results:**

The central loop of H-reflex was (6.77 ± 0.28) msec and the sacral cord conduction delay was (1.09 ± 0.06) msec.

**Conclusion:**

The sacral cord conduction time was estimated to be about 1.09 msec in this study and because at least 1 msec is required to transmit the signal across the synapse between the sensory ending and the motor cell, so this estimated time was sufficient for only one central synapse in this reflex.

## Background

The H-reflex was first described by Hoffmann in 1918 [1]. The major clinical application is evaluating the status of the peripheral nervous system with respect to proximal peripheral nerve conduction and potential entrapment of the S1 nerve root. The traditionally performed H-reflex has a very long pathway, reducing its ability to localize a lesion to the S1 nerve root. To overcome this obstacle, H-reflex studies were devised by stimulating the S1 nerve near the first sacral intervertebral foramen by Pease and coworkers [2,3] which was further investigated by others [4-6].

Despite the agreement on its usefulness, there is controversy regarding the synapses involved in its reflex arc. Many authors believe that this is a monosynaptic reflex [7,8]. However, some investigators have hypothesized that the reflex is mainly oligosynaptic [9-11].

In this study, we have tried to estimate the number of the involved synapse(s) in this reflex by calculating the conduction time across the sacral cord, using F-wave and the peripheral and central components of the H-reflex.

## Methods

Forty five volunteers were selected to enrol in this study but due to asymmetric ankle reflex in two of them and inability to record H-reflex in three of them, five subjects were excluded from the study, so the final study group consisted of 22 men (55%) and 18 women (45%).

They had no low back pain and no previous history of neurologic problems, intervertebral disc problem, rheumatic diseases, diabetes and renal or metabolic diseases. It was assured that they had normal symmetrical plantar and achilles tendon reflexes, normal muscle strength, were able to tiptoe and walk on their heels and had no sensory deficit and negative straight leg raising (SLR) test.

After obtaining informed consent, the subjects were examined while relaxed in prone position. Examination was performed in room temperature with their skin warmed to reach normal temperature, if cold.

Dantec 2000C equipment was used. The recording electrodes were surface electrodes with 0.5 cm diameter. The active electrode was placed at the middle of the line connecting the popliteal crease to the medial malleolus. The reference electrode was placed 2 cm distal to it. The ground electrode was placed near the active pick-up electrode over the calves. The stimulator electrodes for stimulation at the popliteal fossa were surface electrodes with 0.5 cm in diameter and cathode-anode distance of 2 cm. The cathode was placed proximal to anode over the tibial nerve. Direct rectangular current pulses were used with a duration of stimulation of 1m sec for H-reflex and 0.2 msec for F and M waves. The stimulation frequency was 0.5 Hz for H-reflex and 1 Hz for F-wave. The amplifier had a filter frequency of 2 Hz to 10 KHz, sweep speed of 5 msec/ division and voltage sensitivity of (0.1–2) mv/division.

The soleus H-reflex was obtained with submaximal stimulation of the tibial nerve at the popliteal fossa. Then peak latency and base to peak amplitude of the H-wave were recorded.

With supramaximal stimulation, F and M waves were recorded. Moreover an averaged F-wave latency, onset latency and base to peak amplitude of M-wave were determined. For determination of averaged F-wave latency, at least 10 F-waves were recorded.

**Figure 1:**
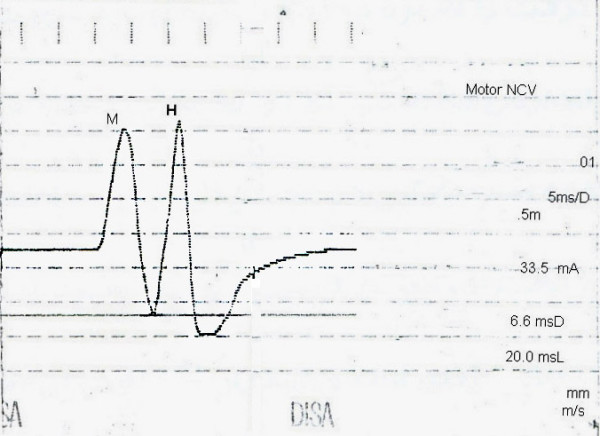
Recording of M and H waves with the S1 root stimulation.

To obtain central H-reflex, stimulation was done using monopolar needle electrode for cathode and disc surface electrode with 0.5 cm diameter for anode. The cathode electrode was inserted at a point 1 cm medial to the posterior superior iliac spine, perpendicular to the frontal plane. After the needle touched the sacrum, it was slightly retracted. The anode electrode was placed over the anterior superior iliac spine. The pickup electrodes were not changed; then, stimulation was applied and increased until the largest H-wave could be seen and peak latencies and base to peak amplitudes of M and H waves were recorded (Figure 1).

Finally, the distances were measured:

-Popliteal crease to the needle.

-Needle to the T12 spinous process.

-Popliteal crease to the T12 spinous

Process (via greater trochanter).

Then processing of the data was done as outlined below:

First, the conduction velocity of the proximal motor and sensory fibers were measured by these formulas:



F* : mean of F onset latencies (ms)

M**: onset latency of M-wave (ms)



Subsequently, conduction time in the afferent and efferent fibers in the central loop of H-reflex was calculated:



We considered the above distance equal to the length of S1 nerve root since the 12^th ^thoracic spine is opposite the first sacral segment [12].

The sacral cord conduction time was calculated with this formula as:

*Sacral cord conduction time = central loop of H-reflex – (delays of the proximal motor & sensory fibers in the central loop)*.

The analyses were performed using SPSS 10.0 software. Independent t-test was applied for statistical analysis of the data. P < 0.05 was considered significant.

## Results

The results of stimulation of the tibial nerve in the popliteal fossa are displayed in Table 1. The results of direct stimulation of the S1 nerve root and recording from the soleus are shown in Table 2. The mean central loop of the H-reflex was obtained as (6.77 ± 0.28) msec. The results of distances between landmarks are summarized in Table 3. Finally, after calculation (as discussed previously), sacral cord conduction time in H-reflex was obtained as (1.09 ± 0.06) msec (Table 4).

**Table 1:** M, H&F waves in the tibial nerve stimulation at the popliteal fossa.

Wave		Mean ± SD
M	Onset latency (msec)	4.3 ± 0.5
	Amplitude (mv)	4 ± 1.6

H	Peak latency (msec)	34.4 ± 2.5
	Amplitude (mv)	1.3 ± 0.7

F	Onset latency(msec)	30.7 ± 1.5

**Table 2:** The M and H waves of the central loop H-reflex.

Parameter		Mean ± SD
M-wave	Peak latency (msec)	19.1 ± 1.7
	Amplitude(mv)	1.4 ± 0.8

H-wave	Peak latency (msec)	25.9 ± 1.8
	Amplitude (mv)	0.9 ± 0.6

Central loop of H-reflex(msec)		6.77 ± 0.22

Intensity of stimulation (mamp)		41.9 ± 8.5

**Table 3:** Distances between landmarks.

Distance	Mean ± SD
Monopolar needle to T12 spinous process (mm)	174 ± 8*
Monopolar needle to popliteal crease (mm)	567 ± 26*
Popliteal crease to T12 spinous process (mm)	742 ± 32*

* Significant difference between men & women (p < 0.0001).

**Table 4:** Conduction times and velocities in central loop of H-reflex.

Parameter		Mean ± SD
Motor fiber	Velocity (m/s)	58.3 ± 2.8
	Conduction time(ms)	2.99 ± 0.15*

Sensory fiber	Velocity (m/s)	64.9 ± 2.3
	Conduction time(ms)	2.69 ± 0.13*

Sacral cord conduction delay (ms)		1.09 ± 0.06

* Significant difference between men & women (P < 0.05).

## Discussion

The H-reflex is perhaps the most extensively studied reflex in the literature on human and mammalian neurophysiology. The relative ease with which the reflex can be elicited in muscles throughout the body, involving both spinal and cranial nerves, has also made the H-reflex an attractive clinical and research tool.

There is controversy regarding the synapses involved in the H-reflex. Many authors believe that this is a monosynaptic reflex arc [7,8]. For instance, Ertekin and coworkers stimulated the tibial nerve at the popliteal fossa and recorded from different lumbar epidural intervertebral levels. The time interval between the negative peaks of the ventral and dorsal root potentials was used to calculate the approximate sacral cord conduction time, which was found to be 1.3 msec. Thus they suggested that the reflex be exclusively monosynaptic [8]. In another study, Ertekin and coworker stimulated the tibial nerve and epidurally recorded the potentials. They proposed that the central conduction time of the soleus H-reflex could be about 1.1 msec[13]. The values of 1.3 and 1.1 msec that were obtained are close to the our value of 1.09 msec.

However, some investigators have hypothesized that the reflex is mainly oligosynaptic [9-11]. For example, Burke, et al. proposed that the rising phase of the increase in excitability of the soleus motoneuron pool produced by electrical stimulation of the tibial nerve lasts more than a few milliseconds and the increase in excitability takes several milliseconds to reach the threshold for motoneuron discharge. Therefore the H-reflex is unlikely to be exclusively monosynaptic[10]. Also, in another study they estimated the duration of the rise times of the excitatory post-synaptic potentials (EPSP) produced in soleus motoneurones by electrical stimulation to be 1.9 msec, so they recommended that H-reflex is not a purely monosynaptic reflex[9].

**Figure 2:**
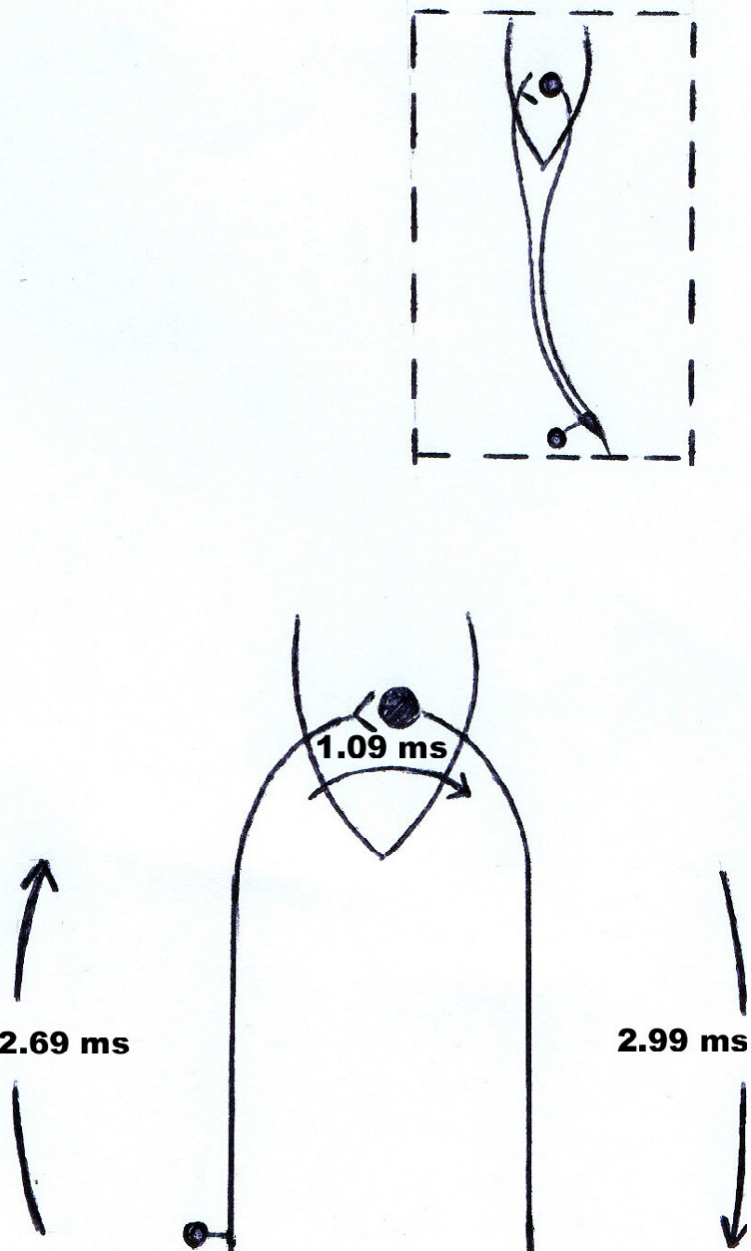
Schematic diagram of conduction time values along the dorsal & ventral roots and in the S1 sacral segment of the soleus H-reflex.

In contrast with some investigators that have hypothesized that there could be some oligosynaptic contributions to the H-reflex [9-11], it can be concluded that the soleus H-reflex is for the most part a monosynaptic reflex, composed of a single synapse between the group Ia fibers and the soleus motor neurons (Figure 2). In this study, the mean sacral cord conduction time was estimated to be about 1.09 msec, so it was sufficient for only one synapse because at least 1m sec is required to transmit the signal across the synapse between the sensory ending and the motor cell [5]. .

As stated earlier, studying the central loop of H-reflex is an important tool to differentiate central from peripheral lesions of the S1 spinal nerve, the normal value of which was (6.77 ± 0.28) msec in this study. This result is in line with the results of previous studies by Pease and coworkers, showing a normal value of (7 ± 0.3) msec[3], by Ghavanini and coworkers with normal value of (6.9 ± 0.4) msec [4], by Zhu and coworkers with normal value of (6.88 ± 0.33) msec [5] and by Sadeghi and coworkers with normal value of (6.78 ± 0.3) msec [6].

Conduction time in the afferent fibers of the central loop H-reflex,(2.69 ± 0.13) msec, and in efferent fibers, (2.99 ± 0.15) msec, were similar to estimations provided by Zhu and coworkers that showed 2.6 msec and 3.2 msec for the afferent and the efferent fibers, respectively [5]. The estimated length of S1 nerve root was (174 ± 8) mm. The resulting value is similar to estimation of Zhu and coworkers in 15 cadavers, that was (175 ± 3) mm [5]. Mean conduction velocity of the afferent (Ia) and the efferent fibers of the H-reflex was (64.9) m/s and (58.3) m/s, respectively. There was no significant difference between men and women in this regard. Its cause seems to be a) longer lower extremity in men and b) more time for proximal sensory and motor fibers conduction in men. (see Tables 3 and 4). Our results are similar to estimations provided by other methods[5,14].

The stimulus intensity to obtain the central H-reflex that had higher amplitude than the central M-wave, was significantly higher than necessary stimulation to obtain the peripheral H-reflex. This more intense stimulation may be due to the fact that the S1 spinal nerve emerges from the sacrum at the anterior aspect. Thus, the needle cannot approach it well from the posterior aspect. Furthermore, the large distance between cathode and anode causes current diffusion, and thereby increasing the best necessary stimulation.

## Conclusion

This study, as many previous investigations, showed that the H-reflex is a monosynaptic reflex arc.

## Competing interests

The author(s) declare that they have no competing interests.

## Authors' contributions

-M.R.A.G : Supervision of the project and helping with calculation.

-A.A. : Data acquisition and testing, writing the paper.

-S.S. : Data acquisition and testing.

-M.R. E : Data acquisition and testing.

## Pre-publication history

The pre-publication history for this paper can be accessed here:


